# In Vitro and In Vivo SERS Biosensing for Disease Diagnosis

**DOI:** 10.3390/bios8020046

**Published:** 2018-05-11

**Authors:** T. Joshua Moore, Amber S. Moody, Taylor D. Payne, Grace M. Sarabia, Alyssa R. Daniel, Bhavya Sharma

**Affiliations:** Department of Chemistry, The University of Tennessee, 1420 Circle Drive, Knoxville, TN 37996, USA; tmoore56@vols.utk.edu (T.J.M.); amoody9@vols.utk.edu (A.S.M.); tpayne18@vols.utk.edu (T.D.P.); drl349@vols.utk.edu (G.M.S.); adanie36@vols.utk.edu (A.R.D.)

**Keywords:** SERS, biosensing, diagnostics, neurological disease, diabetes, cardiovascular disease, cancer, viral disease

## Abstract

For many disease states, positive outcomes are directly linked to early diagnosis, where therapeutic intervention would be most effective. Recently, trends in disease diagnosis have focused on the development of label-free sensing techniques that are sensitive to low analyte concentrations found in the physiological environment. Surface-enhanced Raman spectroscopy (SERS) is a powerful vibrational spectroscopy that allows for label-free, highly sensitive, and selective detection of analytes through the amplification of localized electric fields on the surface of a plasmonic material when excited with monochromatic light. This results in enhancement of the Raman scattering signal, which allows for the detection of low concentration analytes, giving rise to the use of SERS as a diagnostic tool for disease. Here, we present a review of recent developments in the field of in vivo and in vitro SERS biosensing for a range of disease states including neurological disease, diabetes, cardiovascular disease, cancer, and viral disease.

## 1. Introduction

Raman spectroscopy (RS) has gained enormous interest as a biological sensing technique due to its excellent chemical specificity, as it provides a fingerprint-like spectrum without interference from water. RS also employs simple instrumentation with little-to-no sample preparation. Raman scattering, however, is an inherently weak scattering technique. Two methods to enhance the Raman signal employ resonance Raman effects, which provide 10^2^–10^6^ enhancement, or surface-enhanced Raman spectroscopy (SERS), which results in up to 10^8^ enhancement. Combined, resonance Raman and SERS can provide enhancements of up to 10^16^ [1].

In the four decades since the observation of the anomalous pyridine signal on roughened silver electrodes [2] and the subsequent debate over the mechanism of enhancement [3,4], SERS has emerged as a preferred technique when sensitivity and specificity are paramount. It is widely accepted that the enhancement is due to locally amplified electric fields generated when conduction band electrons in metal nanoparticles smaller than the wavelength of the exciting light couple with surface polaritons, and oscillate at a frequency referred to as the localized surface plasmon resonance (LSPR) [5]. Noble metals such as silver (Ag) and gold (Au) exhibit LSPR in the visible and near-infrared regime of the electromagnetic spectrum, due to negative real and small, positive imaginary components of the dielectric functions in this wavelength range. SERS substrates range from colloidal and monodisperse nanoparticles (NPs) of varying morphologies from simple (e.g., spheres [6], rods [7], cubes [8]) to complex (e.g., nanorice [8], nanourchins [9], prisms [8], and polyhedra [8,10]), as well as more complex multidimensional materials that are greatly enhancing and reproducible (e.g., film-over-nanosphere [1], disc-on-pillar arrays [11]). The enhancement on SERS substrates is generated due to the creation of “hot spots”, the junction between two nanostructured features where the electric fields are concentrated. Due to these localized concentrated electric fields, the SERS phenomenon exhibits a strong distance dependence, such that the highest intensity SERS signals are obtained when the target analyte is adsorbed to or within less than 2 nm from the substrate surface and drops off precipitously at larger distances [12,13]. Major advances have been made in the area of in vivo and in vitro sensing techniques due to the implementation of SERS.

SERS sensing methodologies fall into two broad classifications, direct and indirect. Direct sensing relies on the adsorption or binding of an analyte molecule directly to a plasmonic substrate, and the resulting spectral features correspond to the vibrational modes of the analyte itself. In the indirect sensing regime, the SERS signal comes from a reporter molecule (typically a dye or other strongly Raman scattering molecule [14]) instead of the analyte itself. When direct sensing is used, it can be difficult to correlate spectral intensity to concentration because SERS enhancement is non-linear at high analyte concentrations; there is not always a uniform adsorption of molecules on the nanoparticle surface; and inefficient “hot spot” formation, due to wide spacing between particles, results in decreased enhancement. Additionally, direct sensing in biofluids can result in spectra which are difficult to interpret due to enhancement of the components of the fluid itself [15]. Indirect sensing methods can overcome these disadvantages through the use of capture ligands, molecular recognition agents, or antibodies to selectively capture analyte molecules and bring them close to the enhancing surface. Indirect sensing, however, does not probe the molecule of interest itself, so we gain no information about the vibrational modes of the molecule.

Sensing techniques for biological applications must be sensitive enough to measure small concentrations of analyte, and the excellent sensitivity of SERS makes it an outstanding technique for in vivo and in vitro sensing applications. Most disease states start with small changes in cellular processes that become amplified the longer the disease progresses without medical intervention. While there are techniques that have a similar sensitivity, these techniques typically require extensive sample processing and complex instrumentation. SERS achieves comparable limits of detection (LODs) without these prerequisites, and it has been demonstrated that SERS can achieve single molecule LODs in the optimal case of a molecule with an electronic transition near the frequency of the LSPR (resonant), a large Raman cross section, and an attractive electrostatic interaction between the analyte molecule and the substrate [16]. SERS measurements are also non-destructive and minimally- to non-invasive, which makes it a particularly attractive technique for use in in vivo studies.

The World Health Organization (WHO) compiles statistics on the leading causes of death worldwide and, as of 2015, the top 10 causes of death included neurological diseases, diabetes, cardiovascular diseases, cancers, and viral diseases [17]. These diseases will serve as the focus of this review, which is designed to provide broad insight into recent applications of SERS for in vivo and in vitro biosensing. We direct the reader to the literature for in-depth reviews about fundamentals and other applications of SERS, including: the SERS phenomenon [1], hot spot creation and characterization [18], high-performance SERS substrates [19], SERS-based diagnosis [5,15], multimodal [5] and SERS imaging [20], and SERS quantitation [21].

## 2. Neurological Diseases

An understanding of the changes in the concentrations of neurotransmitters in various regions of the brain would aid in the diagnosis and treatment of neurological diseases. In order to detect these changes in neurochemistry, there is a need for a technique with the ability to detect nanomolar concentrations of neurotransmitters with multiplexing capabilities while maintaining good spatial and temporal resolution. SERS has proven to be an excellent candidate for monitoring neurotransmitters and other biological molecules associated with neurological disease with the use of metal NPs. To determine the feasibility of the use of metal nanoparticles in biological systems, toxicity studies have been conducted for AgNPs and AuNPs. Ahmed et al. found that the administration of AgNPs in rats induced brain oxidative stress and alterations in neurotransmitters and amino acids. Astrogliosis and demyelination of neurons associated with neuronal degeneration and vacuolation were discovered in histological examinations. They also found, however, that the concurrent administration of rutin counterbalanced the toxic effects triggered by the AgNPs [22]. The uptake of AuNPs and their use as suitable nanoparticle sensors for intracellular SERS imaging was also studied, and it was found that the cellular uptake of the AuNPs depends on the size of the particles and incubation time. The motility of AuNPs after uptake and processing through the endocytotic pathways was investigated with particle tracking, intracellular diffusion coefficients, and characteristic transport velocities. The results demonstrate that AuNPs are suitable intracellular probes for use in SERS, which takes advantage of the AuNP aggregates in the cells to report their local chemical environments, making them an excellent candidate for in vivo SERS detection [23].

As SERS was initially discovered in combination with electrochemistry, there was considerable interest in the late 1980s and early 1990s for the detection of electrochemically active neurotransmitters with SERS [24,25,26]. After these early papers, however, this interest quickly declined until recently. The resurgence in interest of electrochemical SERS can partly be attributed to the commencement of the Brain Research through Advancing Innovative Neurotechnologies (BRAIN) Initiative in 2013, which has led to an overall increase in neuroscience research. The feasibility of SERS for the detection of amino acid neurotransmitters such as glutamate and γ-amino butyric acid (GABA) was demonstrated with AgNPs, where limits of detection (LODs) of 10^−7^ M for glutamate and 10^−4^ M for GABA in aqueous solution were achieved. These amino acid neurotransmitters are important for neuroendocrine control and are also linked to epilepsy [27]. The detection of choline and catecholamine neurotransmitters including acetylcholine, dopamine, and epinephrine with Ag has also been demonstrated. A SERS substrate was synthesized by AgNP electrodeposition onto tin-doped indium oxide (ITO) using cyclic voltammetry, and size and surface coverage was controlled by changing the number and rate of scans. These substrates exhibited an enhancement factor of 10^7^, and detection limits of 2 μM for choline, 4 μM for acetylcholine, 10 μM for dopamine, and 0.7 μM for epinephrine were achieved [28]. Acetylcholinesterase (ACHE) activity has also been studied using AuNPs to detect acetylcholine after inhibition with carbaryl and paraoxon, two commonly used toxic pesticides which are known to cause memory loss and impairment of neuromuscular functions [29].

In continuing to optimize parameters for SERS experiments, a comprehensive study of the detection parameters for seven neurotransmitters has been conducted by Moody et al. The optimal wavelength for detection with both AgNPs and AuNPs was studied for melatonin, serotonin, glutamate, dopamine, GABA, norepinephrine, and epinephrine. The results demonstrated that for the catecholamine neurotransmitters, the best detection was achieved with AuNPs at an excitation wavelength of 785 nm, while for amino acid chain neurotransmitters, the best detection was achieved with AgNPs at 633 nm [30]. The adsorption characteristics of the molecules with the metal surfaces have also been investigated. Bailey et al. used electrochemistry to study the role of surface adsorption in SERS measurements of neurotransmitters such as dopamine, serotonin, norepinephrine, and catechol. It was found that the molecular structure and surface affinity influence the sensitivity, and the molecules with the strongest affinity for the surface will have the highest signal-to-noise in SERS experiments [31]. Raman and SERS measurements of melatonin [32] and serotonin [33] have been performed along with DFT calculations that focused on the interaction and adsorption of the different parts of the molecule to the Au and Ag surfaces, respectively. The DFT structural parameters and harmonic vibrational wavenumbers found for melatonin correlate well with the experimental data [32]. The adsorption structure of serotonin formed on an Ag electrode surface changes with negative potential shifts followed by complete detachment from the Ag surface [33].

The detection of dopamine is an elusive, yet highly desirable, target due to the association between the loss of dopamine and Parkinson’s disease. SERS measurements have been acquired on rat pheochromocytoma PC12 cells with Au nanopatterned substrates to determine the effects of cisplatin, bisphenol-A, and cyclophosphamide on the level of extracellular dopamine released [34]. Very low LODs for dopamine have been achieved using an AuNR dimer coated with an Ag shell (0.006 pM) [35] and Au core-Ag shell NP-Au nanorod heterodimers (0.02 nM) [36]. Nanomolar LODs of dopamine and serotonin in simulated body fluid have been achieved using a graphene-Au nanopyramid heterostructure platform [37]. Label-free SERS sensors have been employed to study the synthesis of dopamine with the detection of dopamine, norepinephrine, tyrosine, and phenylalanine [38]. The selective capture of dopamine can be achieved using metal nanoparticles modified with a dopamine-selective probe. This capture has been demonstrated with AgNPs modified with iron-nitrilotriacetic acid [39] and via a sandwich assay consisting of Au nanoplates modified with dopamine antibodies as one layer combined with AuNPs encoded with DNA and antibodies as the other layer [40]. The concentration of norepinephrine in dopaminergic cells can be detected using magnetic dynabeads containing NPs, which are loaded with DNA barcodes and antibodies that can sandwich the target molecule [41]. AuNPs functionalized with Raman reporter molecules and antibodies have also been used for SERS detection of the IgG protein [42].

A number of peptides have been isolated from the skin of amphibians including bombesin-, ranatenism-, and phyllolitorin-like peptides. They are classified based on the amino acid sequences of their amidated carboxyl-terminal octapeptide domains. Bombesin contains 14 amino acids and was originally discovered in *Bombina bombina*, a European frog. Two bombesin-like peptides have been identified and characterized in humans, gastrin-releasing peptide (GRP) and neuromedin B (NMB) [43]. These peptides have a variety of biological functions including the secretion of other neuropeptides and hormones, smooth muscle contraction, the stimulation of gastric secretion, and the stimulation of the growth of various tumor cell lines. The adsorption geometry of bombesin and its C-terminal fragments have been studied using SERS with colloidal AuNPs [44] and platinum nanoparticles (PtNPs) [45]. The results show that the L-methionine at the C-terminus determines the bombesin adsorption onto AuNP and AgNP surfaces. On PtNPs, along with AuNPs and AgNPs, the spectra are strongly influenced by the indole ring vibration of the L-tryptophan at position 8 of the amino acid sequence [45]. Tata et al. also used Au coated silicon wafers to demonstrate that L-histidine, L-methionine, and L-tryptophan are responsible for the interaction with the substrate, and the strength of the interaction depends on where the amino acids are in the sequence [46]. The surface geometry of the neuropeptide Y (NPY) and its C-terminal fragments on AuNPs and AgNPs have also been studied. The results show that the NPY^32–36^ C-terminal fragments were involved in the adsorption process on the metal substrate where tyrosine and arginine are mainly responsible for the interaction with the Ag and Au surfaces [47,48]. Peptides with tryptophan in different chain positions were also investigated with SERS and supported by DFT calculations. It was determined that the interaction of these peptides with AgNPs occurs through the carboxylate and amino moieties, while the interaction with AuNPs occurs through the indole ring [49].

SERS is also an optimal technique for multiplexing measurements, where multiple analytes are measured simultaneously. Monfared et al. combined SERS with a partial least squares (PLS) analysis to achieve an LOD of 8 μM for both glutamate and GABA in serum [50]. The multiplexing capabilities of SERS with a dynamic-SERS nanosensor based on patchclamp-like nanopipettes decorated with Au nanoraspberries has been demonstrated through the monitoring of metabolite secretions near living MDCKII epithelial cells where pyruvate, lactate, ATP, and urea were detected simultaneously [51]. The probes were later used to demonstrate, in a single experiment, the simultaneous detection of ATP, glutamate, acetylcholine, GABA, and dopamine. The nanosensor was also placed near cultured mouse dopaminergic neurons where a series of K^+^ depolarizations were performed, and the detection of elevated levels of both ATP and dopamine was achieved [52]. These experiments demonstrate the potential of SERS to monitor neurotransmitter secretion near living neurons. SERS can also be combined with various techniques for multimodal imaging, allowing for the simultaneous measurement of structural and functional information of neurotransmitters. SERS has been combined with MRI/CT imaging by the administration of a single biocompatible contrast drug based on Au-Fe nanoalloys. These Au-Fe alloys do not show any cytotoxicity, can be easily conjugated with thiolated molecules, have long retention times, and accumulate at tumor sites [53].

**Figure 1:**
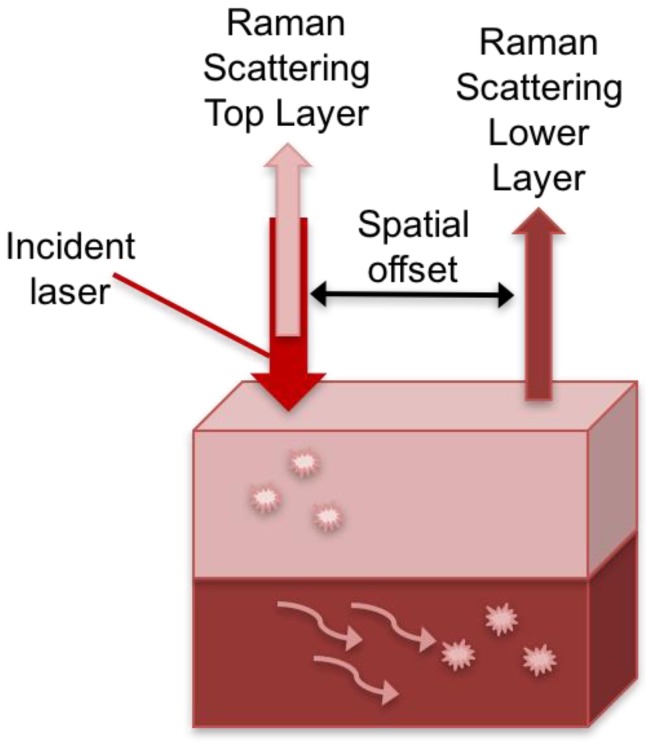
Schematic of spatially offset Raman spectroscopy (SORS) technique. The Raman scattered light collected along the same path as the point of incident illumination is due to the contributions from the surface layer. The Raman scattered light collected at a point spatially offset from the incident illumination point, allows for collection of the signal from subsurface layers where the photons that are deeper in the material migrate laterally before scattering from the surface.

Combining SERS with spatially offset Raman spectroscopy (SORS), termed SESORS, allows for in vivo SERS measurements with increased depth penetration compared to conventional SERS. SORS takes advantage of the spatial component of light where the Raman spectra are collected at a point that is spatially offset from the incident illumination point [54]. Photons that are in deeper layers of material have to migrate laterally before scattering from the surface (Figure 1). Based on this principle, when the light is collected from a point that is spatially offset from the incident irradiation, the ability to probe subsurface layers of material is realized.

**Figure 2:**
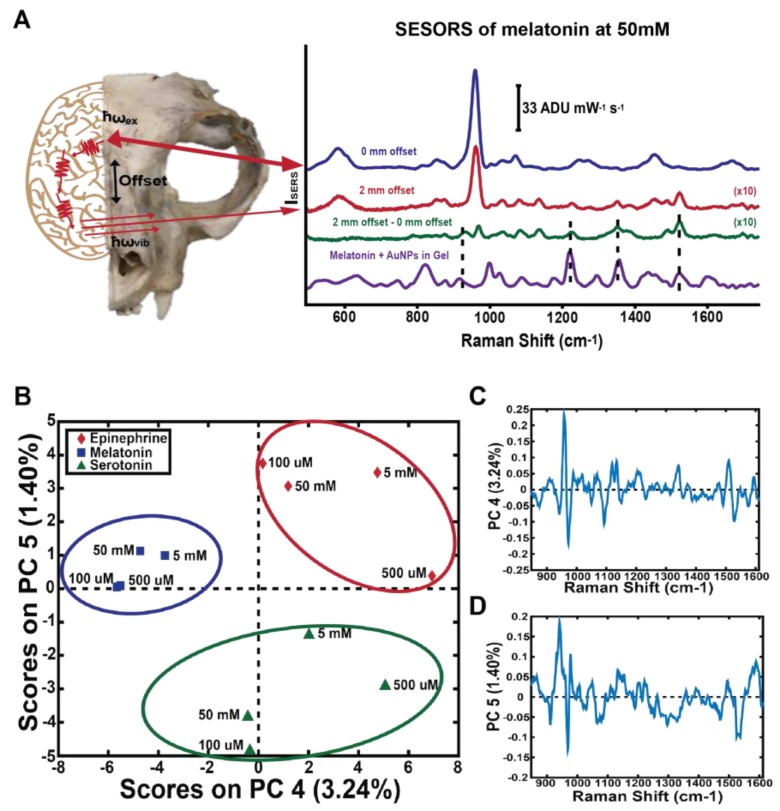
Raman scattered light was collected at a point spatially offset from the incident illumination point, allowing the signal from the neurotransmitter to be acquired through a cat skull (**A**). PCA was used to show that the individual neurotransmitters are clearly separated from each other in a plot of the scores of PC4 versus PC5 (**B**) and their corresponding loadings (**C**,**D**). Adapted with permission from Ref. [55]. Copyright 2017 American Chemical Society.

SESORS was used to monitor neurotransmitter concentrations through the skull, demonstrating the detection of melatonin, serotonin, and epinephrine through a cat skull at various concentrations down to 100 μM [55]. Principal components analysis (PCA), an unsupervised multivariate analysis technique which reduces dimensionality in large data sets, was used to demonstrate that SESORS could clearly detect the individual neurotransmitters through the skull (Figure 2). The PCA analysis also demonstrates that the spectra acquired were unique for each neurotransmitter, resulting in a clear separation of the various concentration spectra into clusters. These results pave the way for potential in vivo neurotransmitter detection through the skull. Coupling SERS with multivariate analysis allows for subtle differences in spectra due to changes between different molecules or normal and disease tissue states to be elucidated, which strengthens the power of SERS for disease diagnosis. Throughout this review, the use of multivariate analysis with SERS will be highlighted to demonstrate the strength of this combination.

## 3. Diabetes

Diabetes mellitus is characterized by the body’s inability to produce or to utilize insulin- a phenomenon that promotes abnormal levels of blood glucose, which patients must consistently monitor to maintain a healthy lifestyle. Proper glycemic management faces certain challenges, not the least of which is accurately detecting small quantities of glucose in the midst of countless other biological molecules, including some with a similar structure. The principal limiting factor in diabetes management is hypoglycemia (<4 mM blood sugar concentration), given that it is especially difficult to monitor [56]. Current technology is incapable of accurately quantifying such low concentrations of glucose [57]. Physicians must be careful not to induce hypoglycemia when administering insulin [58], but they could be more confident in their dosing decisions if sensors with the ability to detect ultralow concentrations of glucose were available. Another concern is minimizing the invasiveness to the diabetic patient during glucose sensing, where biosensors are being designed to extract glucose information from saliva and urine instead of blood. The amount of glucose found in these fluids is much smaller than in blood, which is another reason that novel sensors must have very low LODs. Overall, the main focus of diabetes biosensor development is to obtain accurate, continuous glucose measurements at all relevant concentrations.

There are, notably, 425 million cases of diabetes around the globe [59], and the ability to catalog the temporal fluctuations in blood glucose levels could revolutionize diabetes treatment. Patients would be able to recognize overall trends in their blood sugar imbalances as opposed to simply pricking their finger several times a day to determine if their levels are within the normal range. Continuous glucose monitoring (CGM) sensors have recently become available to patients, but they lack longevity and sensitivity [57,58]. Therefore, researchers are still working towards the development of a long-term, continuous monitor that can sensitively and selectively detect glucose, even at ultralow concentrations.

In recent years, much progress has been made towards the development of a biosensor that uses Raman spectroscopy to monitor diabetes in real time. Dingari et al. and Birech et al. highlight the potential of biosensors that use this method to detect biomarkers such as glycated albumin, leucine, and isoleucine [60,61]. However, due to the small Raman cross-section of glucose, SERS is necessary to combat this weak scattering signal. The progress of SERS biosensor development ranges from preliminary in vitro testing to the successful detection of glucose in urine and saliva and in vivo implementation in animal subjects.

Today, the most commonly used glucose monitors require blood to be drawn from the finger and placed on a strip, where a reaction occurs between the blood glucose and glucose oxidase (GOx), and an electrode measures the intensity of the electric field produced. The newly available CGM sensors exploit the same principle with the modification of the electrode being inserted subcutaneously [58]. The majority of current diabetes SERS-based sensors utilize the catalytic activity of GOx in the oxidation of glucose to gluconic acid and hydrogen peroxide. Qi et al., optimized a substrate coated with AgNPs and functionalized with GOx to act as an indirect glucose sensor chip when excited with a laser [62]. In the presence of more glucose, less SERS signal is obtained because the GOx detaches from the substrate [62]. The advantages of this probe include its high specificity to glucose and that it is non-toxic.

Non-invasive SERS detection of hydrogen peroxide, a product of glucose oxidation, has also been explored as an indirect indicator of glucose concentration. Using a boronic acid probe, Gu et al. have identified a positive correlation between the SERS signal intensity obtained from hydrogen peroxide and the concentration of glucose in artificial urine and human serum [63]. It has been demonstrated that the glucose levels in bodily fluids, including saliva, are proportional to blood glucose levels [64]. Al-Ogaidi et al. have created an ideal SERS substrate for the collection of the hydrogen peroxide signal in bodily fluids besides blood, making use of AuNPs coated with silica, which allows for increased water solubility and also prevents the reaction of gold with peroxide, and then functionalized the silica-coated AuNPs with GOx [65]. This sensor can accurately quantify glucose in vitro in the range from 25 µM to 25 mM and is responsive to the addition of glucose to saliva.

Another focus of SERS diabetes research is the direct detection of glucose. Unfortunately, in addition to the low Raman cross section, glucose exhibits poor adsorption onto metals, making it difficult to take advantage of surface enhancement [62]. In order to successfully bind molecules to transition metal nanomaterials, many researchers employ capture strategies in which molecular recognition agents are functionalized onto a SERS-active substrate and are then used to bind target molecules to bring them within the enhanced sensing volume. Most commonly, these molecular recognition agents are antibodies specific to the analyte of interest, but it is also possible to use aptamers, nucleic acid sequences, and other molecules with chemistries that help preferentially select an analyte. Ma et al. have sought to improve upon continuous monitoring technology by designing an implantable SERS substrate composed of Ag-coated nanospheres with a surface layer of decanethiol/6-mercapto-1-hexanol (DT/MH) as a capture strategy to detect glucose using SESORS [66]. Commercially available continuous monitors are unable to accurately track glucose levels for more than a week, require multiple calibrations each day, and cannot accurately measure the low concentrations of glucose that are characteristic of hypoglycemia [66]. The DT/MH sensor directs glucose to the surface of the nanospheres and enhances its Raman signal. Using SESORS for through the skin measurements in rats, the sensor has been shown to provide an accurate detection of hypoglycemic levels of glucose and to function well for a minimum of 17 days. One critique of the DT/MH capture layer is that it is not specific to glucose but can capture many other diol-containing molecules [62,67].

Alternatively, boronic acids, which are widely accepted as capture molecules for saccharides [68], can be modified to achieve a high selectivity for glucose binding. The geminal hydroxyl groups of boronic acids eagerly bind to the cis-diols of various sugar molecules; however, not specifically glucose. Sharma et al. incorporated scaffolding into the synthesis of a bisboronic acid capture ligand composed of two 4-amino-3-fluorophenylboronic acid molecules for the direct, selective sensing of glucose [67]. Using multivariate analysis, they were able to differentiate between normal (4–8 mM), hypoglycemic (<4 mM), and hyperglycemic (>8 mM) glucose levels in vitro [67].

**Figure 3:**
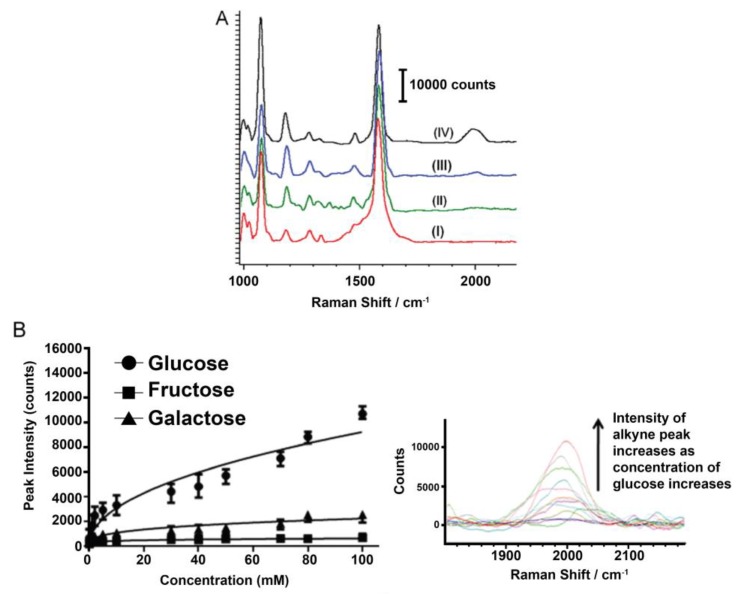
(**A**) Comparison of SERS spectra of phenylboronic acid-functionalized substrate with (I) no analyte, and 10 mM of (II) fructose, (III) galactose, and (IV) glucose; (**B**) SERS intensity of alkyne peak at 1996 cm^−1^ with various concentrations of glucose, galactose, and fructose. Inset is the Raman spectra, showing the alkyne peak with various concentrations of glucose. Reprinted from Ref. [70], Copyright (2014), with permission from Elsevier.

Other researchers rely on bidentate complexes that sandwich glucose molecules between boronic acids and label molecules with strong, distinct Raman peaks in regions with little biological interference. For instance, Kong et al. have designed a Au and Ag coated substrate functionalized with a boronic acid molecule to capture glucose, along with other sugars, and label it with a metal carbonyl that exhibits a preference for glucose with a characteristic SERS peak at 2111 cm^−1^ [69]. They demonstrated, in vitro, the gradual increase in SERS signal with an increase in the amount of glucose, as well as the accurate quantification of 5 mM glucose in urine. In a later study, Kong et al. proposed a modification of their previous sensor that replaced the metal carbonyl label with an alkyne group, which exhibits a characteristic peak at 1996 cm^−1^ [70], and the results are summarized in Figure 3. This sensor is cost effective due to its reusability after being washed with a mildly acidic solution.

Moreover, Gupta et al. have developed a more complex substrate for direct glucose detection that makes use of several of the aforementioned techniques [71]. The process involves coating AgNPs with an Au shell, then GOx, followed by mercaptophenyl boronic acid to selectively capture glucose and acquire the SERS signal. A linear calibration curve was obtained through in vitro measurements in the range from 2 mM to 6 mM, and the accurate quantification of glucose was achieved in blood samples. The sensor was determined to be stable for at least 30 days and provide selectivity for glucose when combined in a matrix consisting of potentially interfering proteins.

It is also important to note the recent innovations in in vitro SERS methods for diabetes diagnosis. By performing PCA on the SERS spectra obtained from forty urine samples, Zou et al. were able to discern between diabetic and healthy patients [72]. The differences in the intensity of certain peaks between the two groups correspond to known phenomena, such as the smaller quantity of uric acid or the greater amount of albumin present in the biofluids of diabetic patients. Additionally, Lin et al. have used SERS to document how the structure of oxyhemoglobin changes with the development of diabetes [73]. The spectral variations of the blood samples allow the group to identify diabetic patients versus healthy patients.

From the direct detection of glucose by boronic acid capture ligands, label molecules, and sandwiching to the indirect sensing of glucose through its catalyzed oxidation, there are many opportunities to implement SERS in the clinical management of glucose levels. These techniques are likely to advance the longevity, scope, and convenience of continuous glucose monitoring technology and improve the lives of diabetic patients.

## 4. Cardiovascular Disease

SERS is very effective for the identification and quantification of biomarkers of cardiovascular diseases, such as uric acid. Pucetaite et al. utilized Fourier transform (FT)-Raman to detect uric acid with colloidal SERS substrates [74]. Three varieties of AgNPs were prepared: citrate reduced, hydroxylamine hydrochloride reduced, and Ag seed-catalyzed ascorbic acid reduced. The hydroxylamine hydrochloride reduced nanoparticles resulted in the greatest SERS enhancement. One issue with this detection method, however, is the difference between the band positions in the SERS spectrum of uric acid on the hydroxylamine hydrochloride AgNPs compared to the normal Raman spectra, creating difficulty identifying uric acid in biological fluids. The greatest changes observed are a result of uric acid molecules tautomerizing/deprotonating from interactions with the silver surface and in the aqueous solution.

C-reactive protein (CRP) is another marker that has recently been studied as a potential identifier of cardiovascular disease, using an AuNP-based SERS bioassay for detection [75]. In this bioassay, AuNPs are functionalized with an antibody for CRP, along with horseradish peroxidase (HRP) and leucomalachite green (LMG). LMG is not a strong Raman scattering molecule; however, when exposed to H_2_O_2_ through the conjugated HRP, it is reduced and converted to malachite green (MG), which exhibits a strong SERS signal. The MG SERS signals changed in intensity in response to varying concentrations of CRP, which suggests that the assay has positive sensitivity and selectivity for detecting CRP. N-terminal pro-brain natriuretic peptide (NT-proBNP) is a sensitive cardiac biomarker for heart failure, and has been detected with SERS using a sandwich assay [76]. The sandwich assay was comprised of CoFe_2_O_4_ nanoparticles functionalized with AuNPs and antibodies as one layer, and microspheres of metal-organic-framework (MOF) functionalized with Au tetrapods, Toluidine Blue reporter, and a second antibody as the second layer. This sandwich assay demonstrated high selectivity and specificity for NT-proBNP over other possible interferents, while achieving an LOD for NT-proBNP in the sub-fg mL^−1^ concentration range.

Myoglobin, a cardiac biomarker that is released within one hour after the appearance of chest pain during acute myocardial infarction (AMI), has been detected using Ag nano-pinetree substrates in both aqueous and urine samples with a wide linear dynamic range (up to 5 μg mL^−1^) and an LOD of 10 ng mL^−1^ [77]. Blood plasma has also been analyzed using colloidal AgNPs to detect AMI through the detection of several biomarkers consistent with a coronary event. When the SERS results were analyzed using a combined PCA-linear discriminant analysis (LDA) method, this produced a model with 87.5% sensitivity and 93.8% selectivity for distinguishing the serum from patients with AMI vs. those without [78]. Very recently, a plasmonic chip substrate consisting of Au islands on a glass slide has been developed for the detection of a gene related to AMI consisting of 21 DNA bases that is over-expressed in the serum of patients with a genetic predisposition to AMI [79].

Platelet-derived growth factor (PDGF) is a mitotic factor that contributes to the development of atherosclerosis and coronary heart disease (CHD) and exists as a series of dimers, of which the PDGF-BB dimer has been demonstrated to act as a biomarker for CHD [80]. Using Ag colloids and urine samples from 87 CHD patients both with and without percutaneous coronary intervention (PCI) and 20 healthy human urine samples, Yang et al. were able to directly identify a characteristic peak (1509 cm^−1^) corresponding to the presence of PDGF-BB, and using PCA-LDA, constructed a model for diagnosis that had 87.9% sensitivity and 87.0% specificity using the PDGF-BB peak, showing the great potential of SERS as a diagnostic clinical technique for CHD.

An SERS immunoassay demonstrates the multiplexed detection of creatine kinase isoenzyme MB (CK-MB) and troponin I (cTnI), two sensitive and selective biomarkers for AMI [81]. The indirect sandwich-style immunoassay consisted of two sets of magnetic capture beads, each functionalized with monoclonal antibodies specific to either CK-MB or cTnI, as well as SERS nanotags functionalized with one of the monoclonal antibodies and with Raman reporter molecules. Using this method, LODs were found to be 42.5 and 33.7 pg mL^−1^ for CK-MB and cTnI, respectively. When compared to an electro-chemiluminescent assay, the SERS-based immunoassay showed a 100 to 1000 times higher sensitivity, which shows great promise in the early diagnosis of AMI. A SERS-based lateral flow immunoassay (LFIA) has also been developed to quantify CK-MB and cTnI, wherein AgNPs were functionalized with Nile Blue A (NBA) prior to use as seeds for the synthesis of Ag-NBA@Au core-shell NPs [82]. LODs for this LFIA were determined to be 0.44 and 0.55 pg mL^−1^ for cTnI and CK-MB, respectively, which is well below the clinical cutoff values. This immunoassay also quantified myoglobin, another important cardiac biomarker, with an LOD of 3.2 pg mL^−1^ [82]. This assay shows an increased sensitivity to a previous SERS-based LFIA that used thiol-coated Au@glass core-shell NPs, where the LODs for cTnI and CRP were established in the μg mL^−1^ range [83].

**Figure 4:**
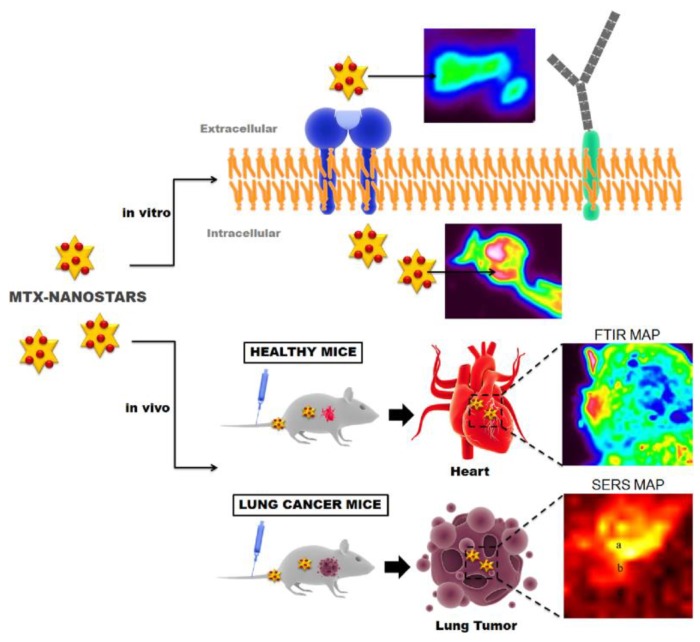
Illustration of the use of mitoxantrone (MTX) nanostars for in vitro and in vivo imaging, as well as nonspecific therapy for patients with cardiovascular diseases. Reprinted from Ref. [84] Copyright (2016), with permission from Elsevier.

In vivo SERS detection methods have been developed to diagnose and aid with drug delivery in patients with cardiovascular diseases [84]. Healthy mice and mice with lung cancer were administered gold nanostars conjugated with Mitoxantrone (MTX-nanostars), an anticancer drug, which has also been used successfully as a non-specific anti-inflammatory therapy for patients with cardiovascular diseases or chronic heart failure, creating a SERS active probe. Delivery of the drug was tracked in real-time using SERS detection and Raman/FTIR imaging. This process is summarized in Figure 4. In the healthy mice, in vivo SERS detection found that the MTX-nanostars accumulated in the heart. In vivo SERS spectra of the tumors in the mice with lung cancer also showed characteristic SERS peaks associated with aromatic stretching of C-C (1300 cm^−1^) of the MTX-nanostars. While initially developed as a method for detecting drug delivery to tumors, the MTX-nanostar probes have also been applied to detection of cardiovascular diseases due to the non-specific accumulation of the MTX-nanostars in the hearts of healthy mice.

Atherosclerosis is a cardiovascular disease which causes plaque to build up in arteries. SERS has been used for both treatment and early diagnosis of this condition [85]. Vascular cell adhesion molecule-1 (VCAM-1) is believed to play a role in the development of atherosclerosis, so the detection of VCAM-1 could be used to diagnose atherosclerosis even before clinical symptoms arise. SERS active AuNPs were functionalized with 4-mercaptobenzoic acid (4-MBA) and the 4-MBA spectrum was observed in human coronary artery endothelial cells after VCAM-1 was upregulated, showing the utility of this SERS nanoprobe for biomarker detection.

## 5. Cancer

Cancer is a broad term for diseases in which abnormal cell growth (tumor) is present in a part of the body. If the tumor stays localized, it is classified as benign, with no adverse health effects; however, if the tumor starts to migrate to surrounding tissues and organs, the tumor is classified as malignant. In extreme cases, during this malignancy migration, tumor cells can intercept blood vessels and be transported to further regions of the body to start dividing and growing again, a condition called metastasis.

In 2015, the WHO reported that 8.8 million (about one in six) deaths around the world were due to cancers in general, making cancers the second most common cause of death worldwide, even though 30–50% of cancers are preventable with lifestyle adjustments [17]. It is estimated that in 2010, the economic impact (treatment and time lost from work) from cancer was over $1 trillion USD worldwide. Economically disadvantaged countries experience disproportionate mortality, with 70% of deaths occurring in middle- to low-income countries [17]. Increases in cancer diagnoses over the next two decades are estimated to climb as high as a 70%, resulting in a serious initiative towards finding methods that enable the detection of cancers in early stages, when treatment is most effective.

SERS is an attractive target for implementation in cancer diagnosis due to high sensitivity, little-to-no sample processing, and rapid detection times. The most fatal cancers in men include lung, liver, stomach, colorectal, and prostate cancers; for women, the most fatal cancers are breast, lung, colorectal, cervical, and stomach cancers. There has been significant research interest in the incorporation of SERS techniques into the detection of cancer cells in serum, the monitoring of drug delivery to tumors, and the evaluation of excision margins during the surgical removal of tumors. Herein, we direct our initial focus on recent advances in SERS diagnosis of cancer in the framework of breast, lung, colorectal, and prostate cancers, before turning our attention to recent advances in SERS implementation for other cancer types.

### 5.1. Breast Cancer

Currently, a broad focus of numerous research studies is decreasing the detection limit of tumor cells, including circulating tumor cells (CTCs) in the blood stream and at the excision margins created during tumor removal. Using core-shell plasmonic nanorods, SERS was used to detect and identify the presence of 20 MCF-7 breast cancer CTCs in a 1 mL volume of a blood serum mimic, which contained HeLa (cervical cancer) and HEK (human embryonic kidney) cells [86]. Mucin 1 (MUC1), a glycoprotein which lines the surface of epithelial cells, has been used as a target for aptamer-based capture strategies, since the overexpression of MUC1 is associated with several cancers, including breast cancer. Nanoparticles functionalized with MUC1-specific aptamers have been employed in various studies to detect as few as 10 MCF-7 cells in a 1 mL volume of phosphate buffered saline (PBS) [87], and also to differentiate between mice with MUC1 positive and MUC1 negative tumors [88]. Here, the MUC1 positive tumor demonstrated uptake of the aptamer-functionalized nanoparticles, while the MUC1 negative tumor did not uptake the nanoparticles, which allowed for detection of the MUC1 signal with SERS upon ex vivo analysis. LODs of 0.2 cells mL^−1^ for MCF-7 cells have been achieved using Au nanowire sensors for the detection of telomerase activity, which is hypothesized to be a good target for early cancer detection [89]. Telomerase is involved in the maintenance of telomeres, or DNA protein complexes found at the end of chromosomes, which are key to the survival of cancers. For in vivo detection of MCF-7 cell-based tumors in mice, Au nanobipyramids functionalized with 2-naphtalenethiol as a Raman reporter were used to detect cells to a lower limit of 5 cells mL^−1^, demonstrating the utility of SERS as a diagnostic technique [90].

**Figure 5:**
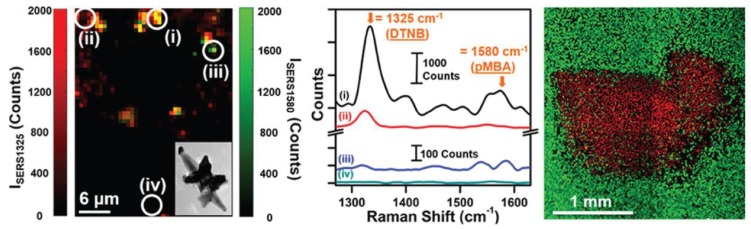
(**Left**) Raman intensity plot of I_SERS1325_ and I_SERS1580_ obtained from multiplex imaging of MDA-MB-231 TNBC cells using multibranched gold nanoantennae (inset) showing signals from (i) both probes, (ii) DTNB alone, (iii) 4-MBA alone, and (iv) no signal; (**middle**) the Raman spectra obtained from (i) both probes, (ii) DTNB only, (iii) 4-MBA only, and (iv) no probes or intracellular lipids; and (**right**) live cells (green) and dead cells (red) observed via confocal fluorescence imaging after photothermal cell death. Reprinted with permission from Ref. [92]. Copyright 2017 American Chemical Society.

An SERS-based method termed Raman-encoded molecular imaging (REMI) has been developed to allow for the rapid, sensitive, and specific determination of cancer cell-positive surgical margins during tumor removal procedures. REMI relies on the topical application of SERS active nanoparticles functionalized with antibodies that are specific to a panel of cell-surface biomarkers, which are obtained from excised lumpectomy and mastectomy tissues. The antibodies used to target the biomarkers include human epidermal growth factor receptor 2 (HER2), membrane estrogen receptor (mER), epidermal growth factor receptor (EGFR), and CD44 [91]. EFGR is overexpressed in the cells of triple-negative breast cancer (TNBC), a highly lethal and aggressive form of breast cancer, and thus can be used as a biomarker for the detection of TNBC cells. Webb et al. have demonstrated a multiplexed detection of both EFGR and programmed death ligand 1 (PGL1), which is also overexpressed in multiple sub-lines of breast cancer cells [92]. To accomplish the multiplexed detection, two distinct Raman reporters 4-mercaptobenzoic acid (4-MBA) and 5,5-dithio-bis-(2-nitrobenzoic acid) (DTNB) were employed, with each reporter conjugated to a multibranched nanoantenna substrate and antibodies specific to the biomarker of interest. SERS detection of the cancerous cells was coupled with photothermal therapies to trigger cell death within the laser spot. A summary of this method is shown in Figure 5.

Cancer cells demonstrate altered glycosylation mechanisms, allowing glycan structures to be investigated as potential biomarkers for cancer diagnosis. Increased levels of sialic acid are concurrent with breast cancer disease states, and this elevation in sialic acid levels has been detected by SERS analysis [93]. Using citrate-reduced Ag colloids, SERS calibration curves for three characteristic vibrational modes of sialic acid (1002, 1237, and 1391 cm^−1^) were measured and used to interpret SERS spectra of saliva samples from healthy individuals and breast cancer patients. It was demonstrated that there is a >5-fold increase in the mean salivary sialic acid level between breast cancer patients and healthy individuals, with the test having 94% sensitivity and 98% specificity [93].

Direct detection of breast cancer markers in serum has been demonstrated for luminal A type breast cancers, which are estrogen-receptor positive, HER2 negative, and Ki67 low; the protein Ki67 can be used to track cell proliferation and high levels of Ki67 are associated with negative outcomes [94]. By coupling SERS measurements with PCA-LDA, a model was generated that could not only diagnose luminal A breast cancer but could also differentiate between cancers at different stages (pT1N0 and pTxN+), with improved performance at the detection of early stage cancers (pT1N0) than advanced cancers (pTxN+).

SERS has been incorporated into immunohistochemical analysis using heat- and protease-induced epitope retrieval (HIER and PIER, respectively) to assist in the staining of human breast tissue blocks. Zhang et al., developed a sandwich style immunoassay consisting of Au nanostars functionalized with secondary antibodies and a fluorescent dye, Alexa 647, as a reporter molecule (Au star-A647G@M). Formalin-fixed paraffin-embedded breast tissue samples were treated with either HIER or PIER and then incubated with anti-HER2 primary antibodies, followed by incubation with the Au star-A647G@M complexes. Both SERS and fluorescence signals from the membranes of cancerous tissue were detected, while normal tissue exhibited neither signal. The antigen retrieval method also made a significant difference, as HIER induced a non-specific adsorption of the antibody in the tissues; PIER does not show this non-specific adsorption [95]. A similar antibody-based SERS sensing approach was applied to track the distribution of nanoparticles on the surface of MDA-MB-435 breast cancer cells. Hybrid gold nanorods with a europium-doped calcium molybdate core were functionalized with the anti-HER2 antibody and Prussian blue dye, as a Raman reporter molecule, allowing for the detection of these cancer cells while non-specific binding interactions were minimized on non-cancerous cells [96].

Recently, SESORS (Figure 1) was used to demonstrate the viability of SERS for in vivo breast cancer detection. MCF-7 cells were incubated with chalcogenpyrylium dye-labeled AuNPs. The functionalized AuNPs accumulated in the cancer cells. The cells were then used to grow multicellular tumor spheroids (MTS), a live breast tissue model which more accurately represents the in vivo 3D tumor environment. The functionalized AuNPs were evenly distributed throughout the MTS. The MTS were placed under 15 mm thick porcine tissues to mimic human skin and the SERS signal of the chalcogenpyrylium dye-AuNPs was detected through the tissue [97]. This again demonstrates the power of SESORS for the in vivo detection of disease.

### 5.2. Lung Cancer

Nanoparticle-antibody complexes using 4-MBA as the Raman reporter molecule were employed to detect EFGR expression on A549 lung cancer cells [98]. Tumors are known to exist in lower pH environments than normal tissue, which make them prime targets for pH-based sensors. pH-sensitive SERS sensors have been developed and implemented as a method to quantify the intercellular acidification of lung cancer cells based on the transformation of the initial substrate, 4-nitrothiophenol adsorbed onto a gold nanorod, under hypoxic conditions to 4-aminothiophenol (4-ATP), which is governed by the pH of the cell [99].

Exosomes are widely accepted as cancer biomarkers. All cell types secrete exosomes, but there are significant differences in the exosomes that are produced by cancerous cells versus normal cells. Park et al. have demonstrated the SERS detection of exosomes from two non-small-cell lung cancer cell lines (NSCLC, lines H1299 and H522) and normal alveolar cells using superparamagnetic Au nanoshell substrates. PCA was applied to uncover differences in the SERS spectra between the normal and cancerous exosomes, and yielded a 93.5% sensitivity and a 97.3% specificity for NSCLC derived exosomes [100]. It was also demonstrated that it is possible for NSCLC cell lines, along with other lung cancer sub-types, to be distinguished from normal cells [100,101]. An ultrasensitive SERS substrate was developed for the detection of multiple microRNA (miRNA) biomarkers for lung cancer using molecular beacons attached to the surface of an Ag nanorod array. This sensor was able to detect attomolar concentrations of the miRNA sequences in human serum, creating the potential for use of this substrate as a clinical lung cancer detection mechanism [102,103].

**Figure 6:**
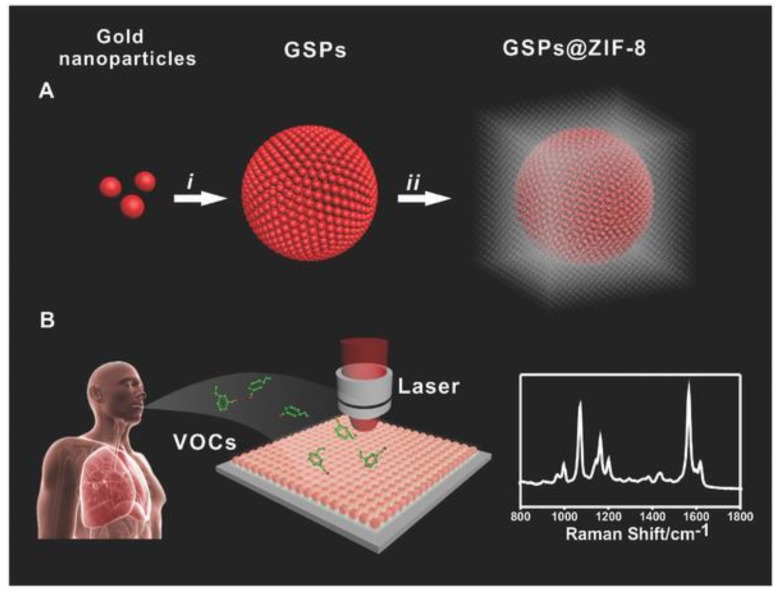
(**A**) Diagrammatic sketch of synthesizing GSP@ZIF-8 core–shell structure: (i) AuNPs assembled into GSPs, (ii) ZIF-8 shell coated on GSP surface; (**B**) volatile organic compound (VOC) detection via SERS spectroscopy. Reproduced with permission from Ref. [104] Copyright 2017, Wiley.

Gas analysis of volatile organic compounds is an emergent strategy for the non-invasive detection of aldehydes that are characteristic of lung cancer. Qiao et al. used a microemulsion assembly method, wherein monodisperse (5.8 ± 0.6 nm mean diameter) AuNPs were assembled into Au superparticles (GSP, 170 ± 30 nm diameter) with single-crystal-like properties. These GSP cores were then functionalized with 4-ATP and coated with a ~150 nm porous ZIF-8 metal-organic-framework (MOF) to provide porosity and preserve electromagnetic fields around the substrate, and this general scheme is summarized in Figure 6. By preselecting the small molecular weight aldehydes via pore size exclusion, coupled with Schiff base reactions between amines and aldehydes, the sensor was able to detect gaseous aldehydes at the 10 ppb level [104]. The use of Ag dendritic nanocrystals functionalized with 4-ATP using Schiff base reactions without the MOF layer has also been tested and resulted in a similar ppb detection of aldehydes [105].

Carcinoembryonic antigen (CEA) is found in over 50% of lung cancer cells and has been detected using an SERS-based immunoassay on live A549 lung cancer cells. The substrates in the immunoassay consisted of Fe_3_O_4_ nanoparticles coated with layers of negatively and positively charged polymers to modify the surface charge and promote the adsorption of AuNPs via electrostatic interactions. The Raman reporter 4-ATP was functionalized onto the hybrid nanoparticle, followed by coupling of an anti-CEA antibody. This immunoassay was shown to be highly sensitive, with as few as 10 cancer cells mL^−1^ detected [106]. This technique was modified further to enable multiplexed detection through the preparation of two different nanotags, containing different Raman reporter molecules and antibodies for either CEA or neuron-specific enolase (NSE). These nanotags were placed in solution with Au-coated magnetic nanoparticles (Fe_3_O_4_, GMNP) and functionalized with antibodies to both biomarkers, creating a sandwich assay with a unique reporter molecule signal for each biomarker [102]. The LODs were reported as 1.48 and 2.04 pg mL^−1^ for CEA and NSE, respectively, demonstrating their potential utility as a clinical tool for lung cancer diagnostics.

### 5.3. Colorectal Cancers

In colorectal cancers, cells extracted from tumors or in circulating blood, as well as exosomes from tumors, are viable targets for diagnosis. SERS, using Ag hydrosols as the substrate, coupled with a PCA-linear discriminant analysis (PCA-LDA), was applied for differentiating between surgically extracted normal cells from healthy individuals and those from rectal cancer patients. The resulting SERS spectra showed decreases in collagen, which arise due to the depletion of cytoplasmic mucin, and increases in metalloproteinase activity in the cancer group [107]. These spectral intensity changes between normal and cancerous cells have potential diagnostic applicability.

Normal Raman scattered light is composed of both parallel and perpendicularly polarized scattered light (with respect to the laser polarization). Using a linear polarizer, the parallel and perpendicularly polarized Raman scattered light can be collected separately. These polarized Raman measurements provide information about the orientation of molecules and the symmetry of the vibrations of the bonds. Polarized SERS has also been explored as a diagnostic technique for colorectal cancers. Blood obtained from healthy volunteers and colorectal cancer patients was analyzed via SERS using a Ag colloid substrate, followed by PCA-LDA analysis. Differences between healthy and colorectal cancer patients’ sera were determined with 91.6% accuracy in both parallel and perpendicular polarizations. The specificity was higher for parallel polarized (93.3%) than for perpendicular polarized SERS (84.4%) [108].

Finally, exosomes, which are vesicles derived from cells that have been identified as possible biomarkers of disease, were analyzed from healthy and tumor-derived colon cancer cells. SERS spectra were collected using silicon nanoarrays, which serve as super-hydrophobic surfaces, decorated with Ag nanograins on the tips of the pillars as the SERS active substrate. It was found that the SERS spectra of normal cells showed elevated lipid amounts, whereas exosomes from tumor cells have increased concentrations of RNA [109].

### 5.4. Prostate Cancer

Prostate specific antigen (PSA) is the most widely used biomarker for the detection of prostate cancer and provides a good target for SERS-based assays. The simplest SERS-based immunoassay for PSA used AgNP-decorated silica cores, labeled with a Raman reporter. The LOD for this sensor was 0.11 pg mL^−1^, which corresponds to femtomolar concentrations. This LOD is well below the 4 pg mL^−1^ threshold value for normal PSA levels [110]. Using an aptamer-based SERS sensing motif, AuNPs functionalized with 4,4′-dipyridyl (DP) and a PSA complementary DNA strand were conjugated to a magnetic NP (MNP) core containing surface aptamers, resulting in an SERS detection limit of 5.0 pg mL^−1^ for PSA [111]. The multiplexing capabilities of SERS have also been applied for PSA, where antibodies for free-PSA (f-PSA) and complexed-PSA (c-PSA) were conjugated to AuNPs labeled with different Raman reporter molecules, malachite green isothiocyanate (MGITC) and X-rhodamine-5-(and-6)-isothiocyanate (XRITC) [112]. PSA is known to complex to other proteins in blood, and some of it binds to a serum protease inhibitor, forming c-PSA. In contrast, f-PSA is not bound to proteins and is lower in men with prostate cancer when compared with men who have benign prostate conditions. Calculating a ratio of the f-PSA versus total PSA (t-PSA) provides a reliable way to identify patients with prostate cancer, particularly in patients who have t-PSA that results in ambiguous results. Using this indirect multiplexed SERS detection, LODs of 0.012 (MGITC) and 0.15 ng mL^−1^ (XRITC) were determined using small volumes (<10 μL) and with short assay times (<1 h). The SERS method resulted in better precision of the f-PSA to t-PSA ratios than other methods, indicating the potential for more accurate diagnoses [112].

The epithelial cell adhesion molecule (EpCAM) is another molecule that is upregulated in prostate cancers, as well as in breast cancers, where it is expressed 100–1000-fold greater than normal cells [113]. Ag-decorated nanorods conjugated with anti-EpCAM antibodies and chemotherapy agents, selectively bound PC3 prostate cancer cells, and enabled the tracking of the nanorod conjugates via SERS microscopy, while simultaneously delivering chemotherapeutics to the cells [113]. This nanorod-antibody-chemotherapeutic drug conjugate was also tested with MCF-7 breast cancer cells (*vida supra*), showing similar positive results.

### 5.5. Additional Cancers

**Figure 7:**
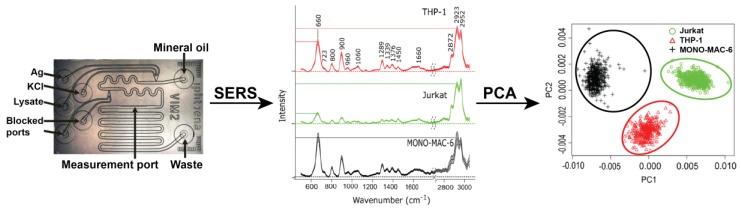
A quartz chip microfluidic device was designed for SERS at 785 nm excitation. At three separate inlets of the microfluidic device, AgNPs, KCl, and the cell lysate are injected and flow to combine together (**left**). Mineral oil is added to the initial mixture to act as a separation medium for the formation of droplets. SERS measurements (**middle**) of the droplets are acquired at a midpoint in the device. PCA of SERS spectra clearly shows separation and identification of the three leukemia cell lysates using variations in band intensity between the spectra (**right**). Figure adapted and reprinted from Ref. [114] by permission from Springer Customer Service Centre GmbH: Springer, Copyright Springer-Verlag GmbH Germany 2017 (2017).

Microfluidic lab-on-chip (LOC) devices, SERS, and statistical analysis have been combined to differentiate between three different leukemia cell lines, cultivated from patients with acute lymphocytic leukemia (ALL, Jurkat cells) and acute myelocytic leukemia (THP-1 and MONO-MAC-6 cells) [114]. SERS spectra were obtained for the three cell lines, followed by PCA on the obtained spectra, resulting in well separated groupings corresponding to each cell type. This process is summarized in Figure 7. A support vector machine (SVM) model, a supervised classification and regression analysis method, was generated to classify the cell types, resulting in mean sensitivities, specificities, and accuracies of >99% for all three cell types, based on >2000 spectra total.

SERS and polymerase chain reaction (PCR) techniques were combined for the tandem multiplexed detection of single-base abnormalities present in melanoma lesions to differentiate between three different point mutations in tumor DNA [115]. This SERS-based genotyping was also applied to mutant and wild type DNA obtained from three melanoma cell lines, with the results confirmed by droplet digital PCR (ddPCR). The SERS-genotyping was then applied to circulating tumor DNA (ctDNA) obtained from melanoma patients, with specificity and sensitivity similar to ddPCR techniques but with simplified PCR strategies [115]. Magnetic core-Au shell NPs functionalized with an antibody to tumor-associated ganglioside GD2, which is expressed in most melanomas, were conjugated onto a graphene oxide sheet for SERS-based detection of circulating malignant melanoma cells [116]. The magnetic core-shell antibody functionalized conjugates allowed separation of the tumor cells from the blood samples through binding of the GD2. As few as 10 malignant cells mL^−1^ were detected using the modified core-shell-graphene oxide system [116].

## 6. Viral Diseases

After the 2009 outbreak of H1N1 (swine flu), research efforts have focused on developing a sensitive and reproducible method for detecting influenza to allow for early detection and thereby avoiding a pandemic. SERS has been used to detect viruses including influenza, Hepatitis, HIV, and those responsible for tropical diseases. A fabricated SERS substrate, consisting of multilayer hybrid Au/Ag nanorods arranged in an array, was conjugated with rhodamine 6G (R6G) and used as a molecular probe. This nanoprobe demonstrated an analytical enhancement factor of ~10^7^ and detection of Influenza A virus strains (H1N1, H2N2, and H3N2) with an LOD of 10^6^ plaque-forming units (PFU) mL^−1^ [117].

Ag nanorod (AgNR) SERS-substrates were developed as a method for detecting genetic markers associated with high pathogenicity in influenza [118]. 5′-thiolated single-stranded DNA oligonucleotides were immobilized on the surface of the AgNR substrates for the detection of synthetic RNA sequence coding for genetic mutation N665, a mutation of the PB1-F2 protein which codes for increased virulence of the flu. Results showed that hierarchical cluster analysis (HCA), another multivariate analysis technique, differentiated between two DNA probes that differed by one base pair with 100% accuracy. Partial least squares (PLS) analysis was used to compare high and low concentrations of binding DNA-RNA, generating an LOD of 10 nM for the developed method. This method was compared to ELISA (LOD 100 nM), demonstrating a one order of magnitude increase in the sensitivity of the SERS-based technique [118].

**Figure 8:**
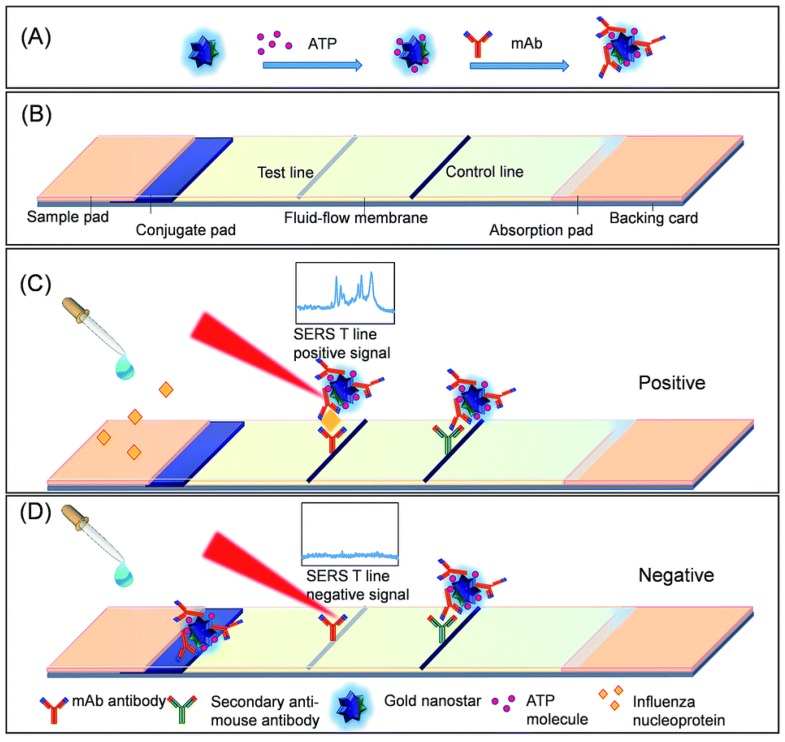
SERS-LFIA test system: (**A**) AuNS is functionalized with 4-ATP and conjugated to monoclonal antibodies to form the SERS tags; (**B**) LIFA test strip, and the response of the SERS-LFIA system to (**C**) influenza A nucleoprotein and (**D**) a negative control. Reproduced from Ref. [119] with permission of The Royal Society of Chemistry.

Maneeprakorn et al. [119] developed a SERS-based lateral flow immunoassay (LFIA), using influenza A nucleoprotein as a target molecule. Au nanospheres (AuNS) were synthesized following the method of Frens [120], followed by the growth of multibranched Au nanostars, which were tagged with 4-ATP (AuNS-ATP)). Antibodies were subsequently conjugated to the AuNS-ATP. Samples containing the target analyte were applied to LFIA strips, where nucleoprotein-NP complexes migrated across the strips to be captured by the antibody conjugated to the NPs and SERS spectra were taken at the site of accumulation on the strip. A summary of this assay is shown in Figure 8. The LOD was determined to be 6.7 ng mL^−1^. Samples of allantoic fluid including influenza A (H1N1) were also tested and supported the validity of the method to test for the virus in a matrix.

It has also been demonstrated that commercially available diagnostic kits for rapid diagnostics of influenza A can show improved detection with SERS-based modifications [121]. AuNPs in a commercial diagnostic LFA kit were replaced with AuNPs tagged with Raman reporter malachite green isothiocyanate (MGITC). The method measured influenza A with high sensitivity, with an LOD of 1.9 × 10^4^ PFU mL^−1^, which was one order of magnitude lower than the commercial LFA kit. This in vitro diagnostic method is a potential solution to the sensitivity and resolution problems encountered when using commercial kits.

Another sensitive influenza virus detection method uses SERS antibody probes that require minimal preparation steps [122]. Citrate-reduced AuNPs were functionalized with the Au binding peptide protein G and a monoclonal antibody to pH1N1 to form a SERS active probe. A capture substrate was prepared with a polyclonal antibody to pH1N1, and the resulting substrate and probes were mixed with pH1N1 virus. The probes were shown to retain their conformation during the antibodies’ reaction with influenza A pH1N1. An enhancing solution of Ag salt and an initiator were applied to the substrate after reaction with pH1N1, and Ag shells were grown around the AuNPs in the probes. Once the Ag surface was prepared, it was functionalized with rhodamine B isothiocyanate (RBITC), and spectra were collected, showing that influenza A could be detected via this method. The LOD for the detection of influenza A pH1N1 was 4.1 × 10^3^ TCID (tissue culture infective dose) mL^−1^. This assay is easily adapted for other targets by simply changing the antibodies on the AuNP surface [122].

One of the most predominant structural features of the influenza virus is its nucleoprotein. Karn-Oracahi et al. [123] have sought to capitalize on this common structural feature by creating SERS probes that consist of AuNPs and labeled with the Raman reporter molecule 4,4-thiobisbenzenethiol (TBBT) and a thiolated carboxylic acid PEG. Following functionalization with the TBBT and PEG, influenza A antibodies were conjugated onto the surface. The SERS assay was created by combining the SERS probes with 2D hydrophilic arrays of Au@Ag core-shell NP substrates. The TBBT served as a molecular beacon and allowed for the detection of selective nucleoprotein-antibody recognition. The SERS immunoassay was then used on complex samples using allantoic fluid with 5.6 × 10^3^ TCID_50_ mL^−1^ of influenza A H1N1 virus. The resulting LOD (6 TCID_50_ mL^−1^) of the substrate showed marked improvement in sensitivity over established LFA methods (fluorescence-based and colorimetric-based).

Hepatitis is a leading cause of liver diseases, including liver cancer, resulting in the need for a detection method. The majority of detection methods often require complex sample preparation and are costly, while surface enhanced methods for the detection of hepatitis B virus (HBV) can be carried out with little to no sample preparation and in a cost-effective manner [124]. A substrate, composed of a silicon chip coated with a polymer brush (poly(MEO_2_-MA)-SH) and AuNPs labeled with the Raman reporter indocyanine green (ICG) as the base, was coupled with a DNA capture strategy to detect HBV DNA in a sandwich assay. After the hybridization of the capture substrate and reporter probes functionalized with DNA/ICG, SERS was used detect HBV at an LOD of 0.44 fM. A sandwich style immunoassay fabricated in tandem with microfluidic devices was developed to detect HBV antigen in human blood [125]. A capture substrate of epitaxially-grown GaN was functionalized with 6-amino-1-hexane-thiol and conjugated to an anti-hepatitis-surface-antigen (anti-HBsAg) antibody. Reporter probes were prepared, consisting of Au nanoflowers functionalized with mercaptosuccinic acid and a new Raman reporter, basic fuchsin (FC), followed by antibody conjugation. The sandwiching capabilities of the two antibody moieties enabled the sensitive detection of the hepatitis B antigen in human blood plasma with an LOD of 0.01 IU mL^−1^.

There is a need for the sensitive detection of human immunodeficiency virus (HIV), which would allow for the earlier diagnosis of HIV infection. This early diagnosis would allow for the start of anti-retroviral therapies prior to severe immune consequences, and detection via SERS has become an attractive method due to the sensitivity afforded by the technique. A SERS-based HIV-1 DNA LFA biosensor, composed of AuNPs labeled with Raman reporter MGITC, was conjugated to thiolated ssDNA complementary to HIV-1 [126]. HIV-1 DNA was quantitatively analyzed with SERS, yielding an LOD of 0.24 pg mL^−1^, and this result was compared with a commercial kit, which had a much higher LOD (80 pg mL^−1^). This SERS-based approach also demonstrated a three orders of magnitude lower LOD than color intensity and fluorescent detection methods.

Mosquito-borne viruses such as zika (ZIKV), West Nile (WNV), and dengue (DENV) have been a topic of research in recent years, due to outbreaks of the diseases. Due to the presence of similar initial symptoms, similar modes of infection, and occurrence in the same geographic regions, it is difficult to distinguish between the two viral mosquito-transmitted tropical viruses ZIKV and DENV. Sánchez-Purrà et al. [127] developed a SERS-based immunoassay with multiplexed detection of the two viral diseases by using SERS-encoded Au nanostars conjugated to antibodies specific to each virus, and with different Raman reporter molecules in an immunoassay to distinguish between nonstructural protein 1 (NS1) biomarkers. LODs for the NS1 protein of ZIKV and DENV were 0.72 ng mL^−1^ and 7.67 ng mL^−1^, respectively. Paul et al. [128] have also reported a bio-conjugated antiflavivirus 4G2 antibody-AuNP based SERS probe in order to detect DENV and WNV. This study showed that the AuNPs can be used as a probe to detect DENV-2 and WNV with an LOD of 10 PFU mL^−1^. This LOD is comparable to those typically seen with quantitative-PCR, but without complex sample preparation and processing. The method was tested against a negative control of mosquito-borne chikungunya virus, with no Raman signal obtained from the negative control. Due to spectral differences between each virus, the method was also able to distinguish between the two viruses.

## 7. Future Directions

SERS allows for the continuous, highly sensitive detection and quantification of various biomarkers and end products of disease states, which makes it an excellent option in the diagnosis and treatment of many diseases that are global public health concerns. With advances in instrumentation and as methods for increasing selectivity towards lowering LODs are developed, SERS will remain an indispensable technique, and continue to demonstrate great potential, for in vitro and in vivo disease detection. The ability to construct substrates from gold, which is non-toxic during in vivo use, is an additional benefit provided by SERS. SERS is likely to advance the longevity, scope, and convenience of continuous glucose monitoring technology, as well as aid in earlier diagnosis of neurological, cardiovascular, and viral diseases, and cancer. As LODs are lowered by the development of more highly enhancing substrates, more sophisticated capture strategies, and multimodal imaging methods, it is apparent that SERS will continue to remain at the forefront of more sensitive analysis in both in vitro and in vivo applications.
